# Plant Chromosome-Specific Probes by Microdissection of a Single Chromosome: Is That a Reality?

**DOI:** 10.3389/fpls.2020.00334

**Published:** 2020-03-25

**Authors:** Fernanda Aparecida Ferrari Soares, Carlos Roberto Carvalho, Mariana Cansian Sattler, Jéssica Coutinho Silva, Denise Eliane Euzébio Pinto, Paulo Zanchetta Passamani, Alex Junior Silva, Wellington Ronildo Clarindo

**Affiliations:** Laboratório de Citogenética e Citometria, Departamento de Biologia Geral, Centro de Ciências Biológicas e da Saúde, Universidade Federal de Viçosa, Viçosa, Brazil

**Keywords:** molecular cytogenetics, chromosome painting, micromanipulation, DOP-PCR, maize, pepper

## Abstract

Painting plant chromosomes through chromosomal *in situ* suppression (CISS) hybridization has long been considered impracticable. Seeking to build specific and complex probes from a single microdissected chromosome, we employed human chromosomes as models to standardize all the necessary steps for application in plants. Human metaphases were used to define the adequate conditions for microdissection, chromosome DNA amplification and labeling through degenerate oligonucleotide-primed PCR, and *in situ* hybridization stringency. Subsequently, these methodologies were applied in the plant species *Zea mays* (chromosome 1) and *Capsicum annuum* (chromosome 7 or 8). The high quality of human and plant cytogenetic preparations and the meticulous standardization of each step, especially the most critical ones – microdissection and first round of DNA amplification – were crucial to eliminate the signs of non-specific hybridization and for direct application in plants. By overcoming these challenges, we obtained chromosome-specific probes, which allowed to achieve a clear and uniform painting of the entire target chromosomes with little or no background, evidencing their complexity and specificity. Despite the high amount of ubiquitous repetitive sequences in plant genomes, the main drawback for chromosome painting, we successfully employed our methodology on two plant species. Both have more than 80% repetitive sequences, which is compared to the human genome (66–69%). This is the first time that plant chromosome-specific probes were successfully obtained from a single A mitotic or meiotic microdissected chromosome. Thereby, we assume that chromosome painting through microdissection and CISS hybridization can now be considered a reality in the field of plant cytogenetics.

## Highlights

We constructed chromosome-specific probes through microdissection of a single plant chromosome. With meticulous steps, the procedure is step-by-step summarized and, from now on, may be applied in other plants.

## Introduction

Chromosome painting is a molecular cytogenetic approach developed for chromosome classification and detection of chromosome aberrations (Pinkel et al., 1986). This molecular cytogenetic approach is based on the painting of individual chromosomes or chromosome regions through fluorescence *in situ* hybridization (FISH). Different from the FISH for a specific DNA sequence, the probe for chromosome painting comprises a cocktail of numerous labeled DNA sequences from a specific chromosome obtained through microdissection or flow sorting (Yang et al., 2016). The DNA from the collected chromosome is then amplified and labeled through polymerase chain reaction (PCR) using a degenerated primer in association with a low initial annealing temperature (DOP-PCR). In such conditions, multiple, evenly dispersed DNA sequences are expected to be amplified, ensuring that the target chromosome is almost completely represented in the probe cocktail (Telenius et al., 1992; Yang et al., 2016). Nonetheless, the specificity of probe hybridization is often hampered by the presence of ubiquitously distributed repetitive sequences. In order to ensure the specific hybridization of the probe with the target chromosome or region, dispersed repetitive sequences must be suppressed, for instance by using an excess of unlabeled, whole genomic DNA, or DNA enriched with repetitive sequences (as Cot-1 DNA). For this reason, chromosome painting is often called chromosomal *in situ* suppression (CISS) hybridization (Lichter et al., 1988; Ried et al., 1998; Sharma and Sharma, 2001).

For humans and other mammals with relatively small genomes, such as rodents and primates (1C = ∼1.5 to 3.0 pg), chromosome painting is well established and has been widely used for clinical diagnosis of chromosome abnormalities (Langer et al., 2004). Besides, chromosome painting has been applied in cytotaxonomic studies to detect chromosomal rearrangements that occur during evolution (Wienberg et al., 1990), and to construct ancestral karyotypes through cross-species *in situ* hybridization (Müller et al., 1999; Rens et al., 2006). Contrastingly, the first painting of specific plant chromosomes was only achieved in 2001 for *Arabidopsis thaliana* (L.) Heynh. (Lysak et al., 2001) after approximately one decade of unsatisfactory results, which were probably due to the large amount of ubiquitous repetitive DNA sequences in plant genomes (Schubert et al., 2001). The successful painting of *A. thaliana* chromosome 4 was facilitated by its small genome size (1C = ∼0.16 pg; Bennet et al., 2003) and relatively low amount of repetitive DNA. In addition, the painting technique involved the application of a pool of BAC clones as probe, allowing to enrich the probe with low-copy sequences (Lysak et al., 2001, 2003). Nonetheless, chromosome painting using DNA probes from flow-sorted or microdissected chromosomes, even associated to the CISS hybridization, has still failed to yield satisfactory results in plant species (Hou et al., 2018). Thus, it is mainly restricted to B or Y chromosomes, most likely due to the presence of chromosome-specific repetitive sequences in these chromosomes, rather than low- or single-copy sequences (Houben et al., 2001). Oligo-FISH has showed to be an alternative for chromosome painting. This approach is based on the selection of thousands of oligonucleotides specific to a target chromosome. However, its application is restricted to species with sequenced genomes (Han et al., 2015; Braz et al., 2018).

Chromosome painting in plant species has another hindrance besides the unspecific signals due to repetitive sequences: the identification and collection of chromosomes. Flow karyotyping followed by chromosome sorting allows the purification of large amounts of a specific chromosome, as long as the chromosome of interest can be distinguished from the other chromosomes based on its optical properties (light scatter, fluorescence; Doležel et al., 2012). In humans the chromosomes are easily discriminated, while in plants the low metaphasic index, difficulties with chromosome release from the cell wall and homomorphic karyotypes are restraints for the successful application of the flow sorting technique (Doležel et al., 2012). On the other hand, probe construction through microdissection is performed by physically collecting multiple copies of a target chromosome or chromosome region (Christian et al., 1999). Usually, a relatively high number of copies (∼20–50) of the target chromosome must be collected, which is considered a limiting technical factor (Christian et al., 1999; Henning et al., 2008). Nonetheless, efforts have been made to adapt protocols in which a reduced amount of target DNA is enough to obtain the chromosome-specific probes (Christian et al., 1999; Henning et al., 2008; Passamani et al., 2018). In plants, the need to dissect more than one copy of the same chromosome is often hampered by the highly homomorphic karyotypes. Therefore, the construction of whole-chromosome probe through the dissection of a single chromosome and amplification through DOP-PCR, which to date has not yet been successfully performed in plants, would be profitable for the progress of plant chromosome painting.

In this work, we present an efficient and reproducible methodology for constructing plant chromosome-specific probes from a single dissected chromosome. Initially, the protocol was standardized in human chromosomes and then adapted and expanded for mitotic and meiotic chromosomes of two different plant species, *Zea mays* L. and *Capsicum annuum* L.

## Materials and Methods

### Biological Material

Human mitotic chromosomes used as cytogenetic model for probe construction were obtained from lymphocytes culture stored in absolute methanol fixative (Merck^®^) at −20°C. The cell bank is maintained in Laboratório de Citogenética e Citometria at Departamento de Biologia Geral (according to Passamani et al., 2018), safety standards and criteria of Ethics in Human Research, Resolution 196/96 of the National Health Council). Seeds of *Zea mays* and *Capsicum annuum* were obtained commercially. The anthers were collected from the flower buds of *C. annuum* cultivated in greenhouses.

### Mitotic and Meiotic Chromosomes

For the germination of *Zea mays* and *Capsicum annuum*, seeds were placed in Petri dishes in growth chambers at 32°C. Roots with a length of 1 cm were incubated for 18 h in 1.75 mM hydroxyurea (Sigma^®^), washed in dH_2_O four times of 15 min each and treated in 3 μM amiprophos-methyl (Sigma^®^) for 4 h. Later, the roots were fixed in 3:1 methanol:acetic acid solution with three changes of 10 min each and stored at −20°C (Silva et al., 2018). Following, the roots were washed again three times in dH_2_O and macerated for 2 h at 35°C in enzymatic pool (4% cellulase + 0.4% hemicellulase + 1% pectolyase, Sigma^®^) diluted in dH_2_O (1:8, enzyme:dH_2_O). After the maceration procedure, the roots were washed in dH_2_O, fixed in 3:1 methanol:acetic acid solution and stored at −20°C.

*Capsicum annuum* meiotic chromosomes were obtained from anthers, which were collected from new floral buds. The floral buds were sequentially fixed in 3:1 methanol:acetic acid, 70% ethanol and absolute methanol, with three changes after 10 min each, and stored at −20°C. The fixed anthers were submitted to two enzymatic macerations. First, the anthers were identified, isolated and macerated in Pectinase (Sigma^®^) for 90 min at 34°C. Next, the anthers were washed in dH_2_O and crushed with a pestle in dH_2_O for meiocytes liberation. Second, a new maceration was performed with 2:5 Pectinase (Sigma^®^):dH_2_O for 90 min at 34°C. Later, the material was fixed with 3:1 methanol:acetic acid for three times of 10 min each and stored in absolute methanol at −20°C.

### Mitotic Chromosome and Meiotic Bivalent Microdissection

For human and meiotic *C. annuum* slides, the absolute methanol of the respective cultures was replaced by fixative solution of 3:1 methanol:acetic acid (Merck^®^). After, the slides were prepared by dropping and air-drying techniques or only by dropping (Caixeta et al., 2011; Arsham et al., 2017) in order to verify how these different strategies influence the dissection of the chromosomes. From the macerated root meristems of *Z. mays*, slides were either prepared by cellular dissociation and air-drying techniques (Carvalho and Saraiva, 1993) or only by cellular dissociation. All slides were checked in a phase contrast microscope Olympus BX41 (Olympus^TM^) using a 40X objective UPlanFl Ph2. Slides were chosen based on the number of prometaphases and metaphases with non-overlapping chromosomes (human and *Z. mays*) or bivalents (*C. annuum*), with preserved chromatin and well-defined telomeres, as well as absence of cytoplasmic debris.

An Eppendorf TransferMan^®^ micromanipulator coupled to an inverted phase contrast microscope IX70 (Olympus^TM^) was used for the microdissection. The slides were immersed in ultrapure water or in 1x phosphate buffered saline (PBS) solution for 1 min, in order to evaluate the hydration effect on chromosomes. The cytogenetic preparations were visualized with an objective LUCPlanFLN UIS 2 60X/0.70 Ph2 and the chromosome microdissections were carried out using sterile Femtotips (Eppendorf^®^) glass microneedles. Relative humidity tests were performed ranging from 40% up to 80% (intervals of 10%) to evaluate the ideal condition for entire chromosome removal.

One human chromosome 2, which was easily identified by its morphology, was collected and used for probe construction standardization. For *Z. mays* and *C. annuum*, one larger mitotic chromosome and one medium bivalent, respectively, were microdissected. Each individual chromosome (human and *Z. mays*) or bivalent (*C. annuum*) was transferred to a sterile microtube of 0.2 mL containing 0.1 μL of sterile collection solution (10 mM Tris–HCl pH 7.5 + 10 mM NaCl + 1 mM EDTA + 0.1% SDS + 0.1% Triton X-100 + 30% Glicerol + 1.44 mg mL^–1^ Proteinase-K, Sigma^®^; Yang et al. (2016). The Proteinase-K treatment was performed for 2 h at 60°C, followed by enzyme inactivation at 80°C for 20 min (adapted from Yang et al., 2016). To avoid contamination, all steps were performed in UV-irradiated biohazard flow chambers. The pipetting procedures were conducted with tips containing sterile filters.

### Amplification of the Microdissected Mitotic Chromosome and Meiotic Bivalent

After deproteinization of each chromosome (human and *Z. mays*) and of the bivalent (*C. annuum*), the DNA was amplified by PCR using a DOP primer (5′-CCGACTCGAGNNNNNNATGTGG-3′; Telenius et al. (1992). The efficiency of amplification was compared between the enzymes USB^®^ Sequenase Version 2.0 DNA Polymerase (Affymetrix^®^), Thermo Sequenase DNA Polymerase (GE^®^), Platinum^®^ Taq DNA Polymerase High Fidelity (Invitrogen^®^) and AccuTaq^TM^ LA DNA Polymerase (Sigma^®^) each for a single chromosome and for a single bivalent. For DNA amplification, programs were adapted from Christian et al. (1999) and Yang et al. (2016), following the parameters recommended for each enzyme and testing the primer annealing temperature, annealing time, ramp between annealing and extension, number of low stringency cycles and extension time. DOP-PCR products were evaluated in 1.5% agarose gel electrophoresis and quantified with NanoDrop^®^ (Invitrogen) and Qubit^®^ (Thermo Fischer Scientific). Again, all steps were conducted in UV-irradiated flow chambers, using sterile pipettes and tips with filter.

### Probe Labeling With Fluorescent Nucleotide and FISH

For the DNA fluorescent labeling we used the random primer method. For this, ten enzymes [Thermo Sequenase DNA Polymerase (GE^®^), Platinum^®^ Taq DNA Polymerase High Fidelity (Invitrogen^®^), AccuTaq^TM^ LA DNA Polymerase (Sigma^®^), Unitaq DNA Polymerase (Uniscience^®^), Pht Taq DNA Polymerase (Phoneutria), AmpliTaq^®^ DNA Polymerase (ThermoFisher^®^), Klenow Fragment (Takara^®^), Hemo KlenTaq^®^ (Bio Labs), Bst DNA Polymerase (BioLabs), and Platinum^®^ Tfi Exo(-) DNA Polymerase (Invitrogen)] were tested in association with the fluorochromes Tetramethyl-rhodamine 5-dUTP (Roche^®^) or ChromaTide^®^ Alexa Fluor^®^ 488-5-dUTP (Life Technologies^®^). The labeled products were visualized in agarose gel electrophoresis 1.5%. For fluorescence contrast, the agarose gel was stained with SYBR^®^ Green (Sigma^®^) for probes labeled with Tetramethyl-rhodamine 5-dUTP (Roche^®^), while agarose gel was stained with Gel Red^®^ (Uniscience^®^) for probes labeled with ChromaTide^®^ Alexa Fluor^®^ 488-5-dUTP (Life Technologies^®^).

For FISH, the slides were prepared according to the “*Chromosome microdissection*” section with the difference that air-drying was applied to all slides. Mitotic *C. annuum* slides were prepared identically to the slides of *Z. mays*. The slides were again selected in accordance to the criteria pointed out above. Hybridization mix was prepared with 200 ng probe + 1.0 ug competitor DNA Cot-1 + 50% formamide + 10% sulfate dextran + 2x SSC. Slides were treated in 1x PBS buffer for 5 min, 4% formalin for 12 min and 70, 85, and 100% cold ethanol series for 5 min each. Chromosome denaturation was carried out in 70% formamide/2x SCC solution at 72°C for 3 min, immediately following 70, 85, and 100% cold ethanol series for 5 min each. The hybridization mix was denatured in a thermocycler at 85°C for 5 min, and immediately transferred to ice for at least 5 min. Then the mix was placed on the slides, covered with plastic coverslip HybriSlip^TM^ (Sigma^®^) and sealed with Rubber Cement (Elmer’s). The hybridization process was performed in ThermoBryte^TM^ (ThermoFisher^®^) at 37°C for 24 h. Later, the stringency washes were performed at 45°C three times in 50% formamide/2x SSC for 5 min each and 2x SSC for 5 min. The slides were mounted in 40% glycerol/PBS + DAPI for chromosomes counterstain, covered with 24 × 40 mm glass coverslip (Knittel Glass) and sealed with nail polish.

### Confirming the Specific Hybridization of the Probes

Slides were analyzed through a photomicroscope Olympus^TM^ BX60 equipped with epifluorescence and an immersion objective 100X/AN 1.4. The images were captured with a digital video camera 12-bit CCD digital video camera (Olympus^®^) coupled to the photomicroscope and a computer with a digitizer plate. Captured images were processed by Image ProPlus 6.1 (Media Cybernetics^®^) and the free software Image SXM.

## Results

### Mitotic and Meiotic Chromosomes

Three different biological materials were used as source of chromosomes: lymphocyte cultures for human mitotic chromosomes, root meristems for *Zea mays* and *Capsicum annuum* mitotic chromosomes, and floral buds for *C. annuum* bivalents. As widely recognized in the literature, plant cytogenetics require larger efforts to obtain mitotic chromosome compared to studies on humans, mainly due to: (a) the lower metaphasic indexes of root meristems, (b) the need to adjust the antitubulinic treatment, and (c) the enzymatic maceration for each species, or even for each root meristem when they show heterogeneous thickness among individuals. With careful preparation of each cytogenetic procedure step (i.e., used compounds, time of each treatment, methods for slide preparation), we were able to obtain slides containing plant cytogenetic material at similar levels to those of human preparations even from different plant source tissues. For meiosis-stage material, the preparation efforts were even more substantial, requiring two steps of maceration (which must be specifically refined) and a laborious search for the anthers with the proper meiotic phase (diplotene and metaphases I, in this paper). Besides, it is worth mentioning that the well-established protocol for obtaining high index of human mitotic chromosomes was fundamental to provide a sufficient number of slides to allow the standardization of the entire procedure, from microdissection to FISH, before its application in plants.

The mitotic chromosomes were obtained from samples of the three taxa by treatment with antimitotic agents. Human lymphocytes, which were previously treated with the antitubulin agent colchicine by Passamani et al. (2018), provided a high proportion of prometaphases/metaphases. For *Z. mays* and *C. annuum*, hydroxyurea (inhibitor of ribonucleotide redutase) was previously applied to arrest root meristematic cells in S phase. After removing the agents through washing, the cells reenter the cell cycle in a synchronized manner. The further exposure of the root meristems to antitubulin, in this case amiprophos-methyl, was efficient in arresting a large number of cells at prometaphase/metaphase. The combination of cell cycle arrest treatment with the slide preparation methodologies (dropping or dissociation with or without air-drying) resulted in widespread chromosomes with well-preserved morphology, different chromatin compaction levels and without overlapping or cytoplasmic background. Due to these features, these slides were considered adequate for microdissection and FISH. The criteria used for slide screening require a careful look at each step of the cytogenetic preparation. This is laborious and difficult, but fundamental for the success of the next steps of chromosome-specific probe construction mainly for plant species.

In this study, *C. annuum* meiotic bivalents at diplotene were obtained from flower buds measuring 3.0 mm in diameter. But a screening of flower buds in different sizes is necessary for each study organism in order to find the desired meiotic phase. The two-step enzymatic maceration and dropping techniques proved appropriate for slide preparation, resulting in widespread bivalents, without overlapping or cytoplasmic background (Supplementary Figure S1).

### Mitotic Chromosome and Meiotic Bivalent Microdissection

The elimination of the air-drying during the slide preparation was a key change to avoid strong chromosome adherence to the slides and to facilitate the microdissection of the mitotic chromosomes (human and *Z. mays*) and meiotic bivalent (*C. annuum*). Slide washes in PBS buffer for 1 min and the relative humidity maintenance at ∼70% provided the most adequate chromosome hydration to allow microdissection without chromatin fragment loss. Supplementary Figure S1). illustrates the microdissection process from meiotic bivalent of *C. annuum*.

### Amplification of the Microdissected Mitotic Chromosome and Meiotic Bivalent

Deproteinization with 1.44 mg mL^–1^ Proteinase-K for 2 h at 60°C resulted in a proper first round of amplification of the target DNA, which we considered one of the critical steps. Amplification conditions that resulted in satisfactory amounts of DNA for the thermostable enzymes Thermo Sequenase DNA Polymerase (GE^®^) and Platinum^®^ Taq DNA Polymerase High Fidelity (Invitrogen^®^) (Supplementary Figure S2A) were: 95°C for 5 min, 10 cycles of 94°C 1 min, 30°C 2.5 min, ramp of 0.1°C/s to 72°C, 72°C for 3 min, 30 cycles of 94°C 1 min, 62°C 1.5 min, 72°C 2.5 min and final extension of 72°C 8 min. For USB^®^ Sequenase Version 2.0 DNA Polymerase (Affymetrix^®^), the amplification condition was: 92°C 5 min, 8 cycles of 25°C 2 min, 34°C 2 min, 90°C 1 min following the second step protocol as described above (Supplementary Figure S2B). Two rounds of PCR for each target DNA using the same enzyme were enough to generate 300 ng μL^–1^ of DNA. The first reaction was performed with 6 μL of final volume, and the second with 50 μL using the total volume of the first reaction. From the second DOP-PCR reaction, the length of the resulted amplified fragments ranged from 100 to 900 bp using Thermo Sequenase DNA Polymerase (GE^®^) and Platinum^®^ Taq DNA Polymerase High Fidelity (Invitrogen^®^) (Supplementary Figure S2A), and 100 to 600 bp using USB Sequenase Version 2.0 DNA Polymerase USB^®^ Sequenase Version 2.0 DNA Polymerase (Affymetrix^®^) (Supplementary Figure S2B). The AccuTaq^TM^ LA DNA Polymerase (Sigma^®^) did not promote amplification of a single chromosome.

### Probe Labeling With Fluorescent Nucleotide and FISH

**FIGURE 1:**
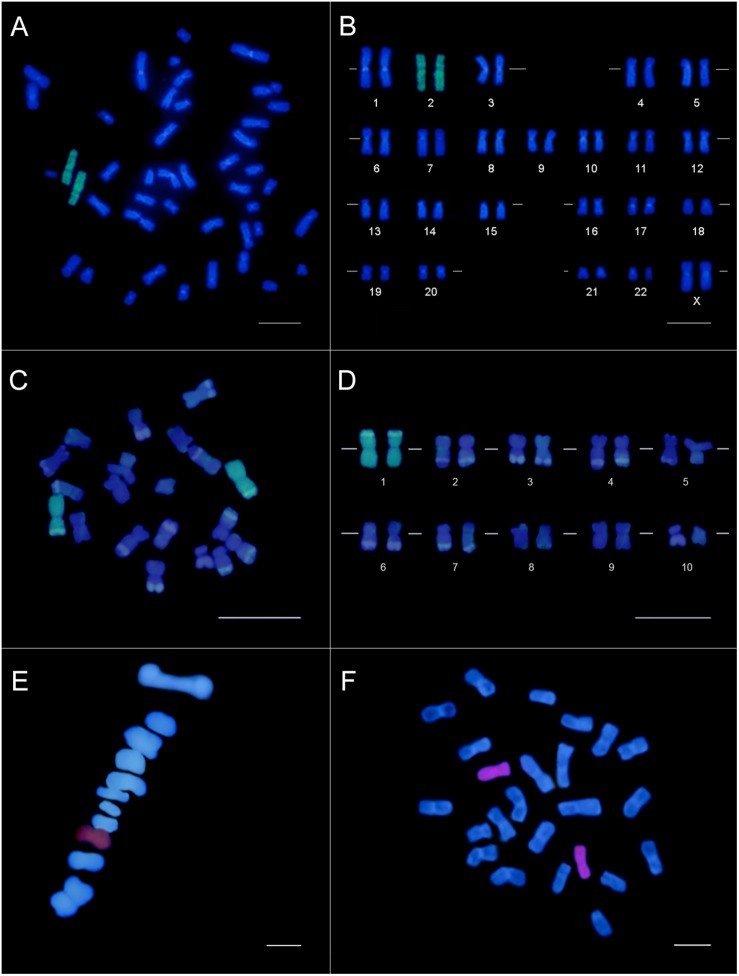
Chromosome painting by CISS hybridization using chromosome-specific probes obtained from the microdissection of a single mitotic chromosome (human and *Z. mays*) or bivalent (*C. annuum*). Human female karyotype **(A)** and karyogram **(B)** showing the specific labeling (green) of the chromosome pair 2. *Z. mays* karyotype **(C)** and karyogram **(D)** evidencing the chromosome pair 1 labeled (green) with the chromosome-specific probe. The DAPI-banding pattern in *Z. mays* chromosomes was caused by this fluorochrome and the denaturation during the FISH, evidencing the knob portions. Bar = 10 μm. A pair (7 or 8) of *C. annuum* metaphase I **(E)** and mitotic metaphasic chromosome **(F)** specifically labeled (red) with the probe constructed from the microdissection of a single bivalent. Bar = 5 μm.

Considering the fluorescent labeling to generate chromosome-specific probes, six out of ten evaluated enzymes incorporated the ChromaTide^®^ Alexa Fluor^®^ 488-5-dUTP (Life Technologies^®^) or Tetramethyl-rhodamine 5-dUTP (Roche^®^) fluorochromes in DNA sequences (Supplementary Table S1) and, consequently, showed a mitotic chromosome or bivalent specific labeling in FISH (Figure 1). The probes obtained from Thermo Sequenase DNA Polymerase (GE^®^) showed relatively high fluorescent intensity signals in agarose gel, which was confirmed by FISH (Supplementary Figure S2C and Figure 1). Meanwhile, the four remaining enzymes did not incorporate the fluorochromes into the DNA sequences and, thus, no FISH signal was observed (Supplementary Table S1).

We obtained a chromosome-specific probe for chromosome 2 of the human karyotype. The FISH stringency conditions of 82% allowed us to observe homogeneous labeling along the entire chromosome, with little to no background (Figures 1A,B). About 95% of the nuclei and metaphases showed specific chromosome labeling, confirming a reproducible and efficient probe construction.

Based on the standardization procedure for constructing the chromosome-specific probe through microdissection in human material, a chromosome-specific probe was obtained for *Z. mays* mitotic chromosome 1 (Figures 1C,D). We also obtained a chromosome-specific probe from a *C. annuum* bivalent, labeling a pair of mitotic chromosomes and meiotic chromosomes in metaphase I (Figures 1E,F). For both plant species, at least 10 labeled prometaphases/metaphases per slides were visualized, demonstrating the probe reproducibility and specificity. Additionally, we observed a labeling efficiency of 65–80% along the slides.

An illustrated guideline for the entire procedure described here is presented on Supplementary Information (Supplementary Figure S3).

## Discussion

Although chromosome-specific probe construction has been considered an extensive and challenging cytogenetic methodology, the standardization of each step allowed us to obtain chromosome-specific probes for human material as well as two plant species. Our research group has been concentrating efforts to improve the chromosome painting technique, aiming especially to reduce the amount of target DNA necessary for the first round of DOP-PCR. To this end, we combined an *in situ* PCR (PRINS) prior to microdissection with the DOP-PCR and successfully constructed a complex probe from ten copies of a small fragment of human chromosome X (Passamani et al., 2018). Advancing in our main goal, the most critical steps were resolved: the chromosome microdissection, the transferring of the chromosome to the microtube and the initial low stringency amplification. By combining the chromosome-specific probes obtained from a single microdissected chromosome with the stringency conditions applied in FISH, the dispersed repetitive sequences were efficiently blocked, and we were consequently able to obtain clear fluorescence painting of the entire target chromosomes. For the first time, chromosome specific probes were constructed from a single microdissected autosomal chromosome of the A complement in two plant species, allowing chromosomal *in situ* suppression (CISS) hybridization to be successfully performed in plants.

Whole chromosome-specific probes were generated for humans, *Zea mays* and *Capsicum annuum* through microdissection of a single chromosome and amplification with DOP-PCR (Figure 1). Although different strategies were used to obtain human and plant chromosomes, the efforts to standardize each step provided morphologically adequate and well-preserved mitotic and meiotic cells, containing individualized chromosomes and bivalents, with well-defined centromeres and telomeres, and with a low level of chromatin condensation. All slides were submitted to cellular dissociation, while air-drying techniques were only applied to the slides submitted to FISH, allowing the chromosomes to be well-spread and flattened on the slide. Although successfully used in plant cytogenetics, the squashing technique has some drawbacks, such as possible morphological deformation of chromosomes, poor spreading of chromosomes on the slide and the eventual loss of biological material when removing the coverslip (Carvalho and Saraiva, 1993). Therefore, we consider cell dissociation and air-drying techniques (Carvalho et al., 2010) fundamental for the successful results obtained through FISH, allowing proper access of the probe fragments to the target DNA immobilized on the slide.

The slides submitted to chromosome microdissection were prepared in the same way as those for FISH, except for the lack of air-drying. The quality of the microdissected DNA is fundamental for the success of chromosome-specific probe construction, being directly dependent on chromosome fixation and staining (Houben et al., 2001). In human cytogenetics, it is common to perform G-banding to correctly identify chromosomes during microdissection. Nonetheless, the staining process is a source of contamination itself and should be avoided in the process of constructing chromosome-specific probes (Christian et al., 1999; Passamani et al., 2018). The high morphological quality of the obtained human chromosomes dismissed the need of staining because chromosome 2 was easily identified. Furthermore, since the main goal of this work was to produce a specific whole-chromosome probe from a single chromosome, the identification of the dissected chromosome was not a requirement. This is especially advantageous for plant species with small and homomorphic chromosomes.

All fixation procedures adopted for the chromosomes to be microdissected should involve a minimal contact with acetic acid, as this substance promotes DNA depurination (Engelen et al., 1998; Houben et al., 2001). Here, the suspension cultures (human lymphocytes and *C. annuum* meiocytes) were fixed in methanol:acetic acid (3:1), but stored in absolute methanol, minimizing the exposure to acetic acid. On the other hand, the root meristems were fixed and stored in methanol:acetic acid (3:1). In our previous attempts, the storage in absolute methanol was not efficient for such biological material, as the cell dissociation was substantially hampered. Nevertheless, for any of the biological materials, the chromosomes submitted to microdissection were not exposed to acetic acid after slide preparation, and the microdissection procedure was performed immediately after obtaining the slides. In an attempt to reduce the contact of the root meristems with acetic acid, we fixed 10 min in 3:1 methanol:acetic acid, then transferred to ethanol 70% and prepared the slides with dH_2_O, as proposed by Zhou et al. (1999). But this fixation condition also hampered cellular dissociation and generated chromosomes with a refringent (shining, vitreous) aspect when visualized in phase contrast, making it difficult to perform the chromosome microdissection.

One of the main features that set the adequate conditions for microdissection is the chromosome hydration. Chromosomes that are too dry become brittle and crumble when microdissected. In contrast, excessively hydrated chromosomes exhibited a gelatinous aspect and do not adhere to the glass microneedle. After washing the slides for 1 min in PBS prior to microdissection, the chromosomes exhibited an ideal hydration, while washing only in ultrapure water resulted in faster drying of the chromosomes. Moreover, the maintenance of the relative humidity of the room around 70% avoided the chromosomes to dry during the microdissection. These strategies facilitated the microdissection of whole chromosomes without loss of visible fragments and ensured the proper chromosome adherence to the glass microneedle and transference to the microtube (Supplementary Figure S1). Engelen et al. (1998) applied a 5 μL drop of ultrapure water over the target chromosomes for rehydration prior to microdissection. Nonetheless, in our experience, this process hampered chromosome visualization and removal due to the water layer. A different strategy was attempted by Christian et al. (1999) that washed the slide in 4x SSC prior to microdissection, but in this case, the excessive amount of salt also hampered chromosome visualization and microdissection.

Enzymatic treatment with 1.44 mg mL^–1^ Proteinase-K for 2 h at 60°C was applied to degrade the chromatin proteins and to liberate the chromosomal DNA before the process of PCR amplification. Passamani et al. (2018) reported a treatment of 24 h with 0.5 mg mL^–1^ proteinase K at 37°C. But despite also being effective to liberate the DNA, the 2 h treatment adapted from Yang et al. (2016) considerably reduced the total time required for probe construction. Each microdissected chromosome was directly transferred to a microtube containing 0,1 μL of the collection solution, minimizing the contamination with exogenous DNA. In approaches like microcloning or ligation mediated PCR, the multiple handling steps involving the microdissected DNA are time-consuming and increase the probability of contamination (Vega et al., 1994). The application of a DOP primer was a remarkable advance in chromosome painting for its simplicity, since there is no need to use adaptors or restriction enzymes, and no previous knowledge of the chromosome target sequences is necessary (Yang et al., 2016).

Fluorescence *in situ* hybridization results showed that the probe pool represented all target chromosome for the three species, even though some DNA sequences might be preferential amplified due to their representativeness in the genome (as repetitive sequences). The painting observed for the three chromosome-specific probes revealed that the DOP-PCR protocol was suitable to generate complex probes. One of the main concerns in chromosome painting is to produce a sufficiently complex pool of probe sequences, which results in a suitable painting along the entire length of the target chromosome (Christian et al., 1999; Yang et al., 2016). For a long time, chromosome painting through CISS hybridization with probes obtained by microdissection was not achievable in plant species (Fuchs et al., 1996; Lysak and Mandáková, 2013; Hou et al., 2018). The main constraint to the success in this technique was the inefficient blocking of dispersed repetitive sequences, which are characteristic of plant genomes (Fuchs et al., 1996). While humans possess around 66–69% repetitive sequences (Koning et al., 2011), the genomes of the plant species used in this work are composed of more than 80% repetitive sequences: ∼81% for *C. annuum* (Qin et al., 2014) and ∼85% for *Z. mays* (Schnable et al., 2009). Slight preferences in primer annealing in the initial low temperature PCR might produce asymmetries during amplification (Christian et al., 1999). Therefore, a high amount of non-chromosome-specific repetitive sequences, which are common in plant genomes (Shcherban, 2015), would favor the generation of asymmetric and poorly representative probes. In addition, the presence of contaminant DNA from other chromosomes due to insufficiently spread metaphases might also contribute to the generation of unspecific sequences in the probe pool (Christian et al., 1999; Guan, 2003). Based on these hindrances, our concern was to associate well-spread and preserved chromosomes with the proposed procedures of microdissection, DNA amplification and hybridization, which allowed us to overcome these issues by generating sufficiently complex and specific chromosome probes.

The three enzymes used for the initial DOP-PCR (Thermo Sequenase DNA Polymerase, Platinum Taq DNA Polymerase High Fidelity and USB Sequenase Version 2.0 DNA Polymerase) amplified the DNA from a single microdissected chromosome. While both Taq produced fragments that ranged from 100 to 900 bp, the fragments generated by Sequenase varied from 100 to 600 pb (Supplementary Figure S2). Nonetheless, some Taq polymerases are not able to amplify the DNA from a chromosome template. Besides, all basic components must be optimized for a successful reaction, mainly the pH of the PCR buffer and the Mg^2+^ concentration (Yang et al., 2016). The initiation is the most critical step of the PCR when a microdissected chromosome is used as target DNA. As the initial annealing temperature is low (30°C), to allow a broad annealing throughout the entire chromosome, thermostable enzymes with optimal temperature around 72°C were initially considered unsuitable for PCR initiation, in accordance with Guan (2003). In our experiments, both Taq polymerases successfully initiated the amplification and provided a larger final concentration of amplified fragments when compared with USB Sequenase Version 2.0 DNA Polymerase (Supplementary Figure S2). Six of the ten different enzymes tested for DOP-PCR labeling successfully incorporated the labeled uridine nucleotides Tetramethyl-rhodamine 5-dUTP or ChromaTide Alexa Fluor 488-5-dUTP. The practicality is the main advantage of this methodology, since the same conditions of the second DOP-PCR amplification step can also be used for labeling (Telenius et al., 1992). Moreover, labeling through PCR allows to restrict probe length to 200–600 pb, which is considered the optimum length for *in situ* hybridization (Morrison et al., 2003).

The probe obtained for human chromosome 2 exhibited a strong hybridization signal with low background (Figures 1A,B) and a high reproducibility (95%). The labeling of *Z. mays* chromosome 2 was consistent with the microdissection of a large chromosome, which was only identified after karyotype assembly (Figures 1C,D). Similarly, the probe obtained for *C. annuum* also showed specific labeling of a single chromosome pair (Figures 1E,F). However, the highly homomorphic karyotype did not allow us to distinguish between chromosomes 7 and 8. Based on these FISH results obtained for the three species, the produced probes were sufficiently complex to allow a uniform painting across the entire chromosome without individual spots. The hybridization efficiency for both plant species was 65–80%, an impressive value considering the well-known difficulties for obtaining suitable preparations in plant cytogenetics. In plants, each species requires a specific and refined adjustment to obtain chromosomes. Consequently, the slides are more heterogeneous regarding the number of prometaphases and/or metaphases and the level of chromatin condensation, which might have contributed to the lower efficiency in hybridization.

## Conclusion

Surpassing the assumptions that CISS hybridization is not feasible in plants, mainly in species with a large number of repetitive sequences, we can here show a pathway (Supplementary Figure S3) starting with the microdissection of a single mitotic or meiotic chromosome. With this, plant chromosome-specific probes from microdissection of a single chromosome have become a reality, representing a new method for painting besides the artificial chromosome clones (BACs/YACs) and amplification of a large number of single-copy sequences. The methodology described here has the potential to be applied in any plant species, with the pre-requisite of high quality cytogenetic preparations to supply chromosomal DNA of similarly high quality. We hope that the application of the methodology contributes to a breakthrough in the field of plant cytogenetics, aiding the development of taxonomic and evolutive studies.

## Data Availability Statement

All datasets generated for this study are included in the article/Supplementary Material.

## Author Contributions

FS, CC, MS, and WC designed the research. FS and MS performed the molecular cytogenetic experiments. DP, PP, and JS performed the classical cytogenetic experiments. All authors analyzed the data and wrote the manuscript, equally contributed to the manuscript edition and revision, and approved the final manuscript version for submission.

## Conflict of Interest

The authors declare that the research was conducted in the absence of any commercial or financial relationships that could be construed as a potential conflict of interest.

## Supplementary Material

FIGURE S1 Microdissection of *C. annuum* bivalents at diplotene stage. **(A)** Bivalent at diplotene. **(B)** Glass microneedle for chromosome microdissection. Two focus planes: the slide and microneedle planes. **(C)** Microneedle removing a bivalent from the slide. **(D)** Chromosome adhered to the extremity of the microneedle. **(E)** Diplotene bivalent microdissected. **(F)** A second bivalent being removed showing reproducibility of the process. Each bivalent was placed in a different microtube, providing the second bivalent sample. The preparation was stained with a 5% Giemsa solution in a phosphate buffer (pH 6.8) for photo documentation only.

FIGURE S2 Representative DOP-PCR products analyzed in 1.5% agarose gel. Lines M represent the base pair marker and lines 1–8 the amplification products resulted of different microdissected chromosomes. **(A)** Human chromosomal DNA (columns 1–3) and *Zea mays* chromosomal DNA (columns 4 and 5) amplified with Thermo Sequenase DNA Polimerase (GE) and Platinum^®^ Taq DNA Polymerase High Fidelity (Invitrogen), respectively. The fragment size ranged from 100 to 900 pb. **(B)** Human (columns 1 and 2) and *Z. mays* (columns 3 and 4) chromosomal DNA amplified with USB^®^ Sequenase Version 2.0 DNA Polymerase, showing fragments size of 100–600 pb. **(C)** Human DNA fragments amplified (columns 2, 4, 6, and 8) and *Z. mays* (columns 1, 3, 5 and 7) labeled with Chroma Tide Alexa Fluor 5-dUTP. Columns 1 and 2 were labeled with AccuTaq^TM^ LA DNA Polymerase (Sigma), columns 3 and 4 with Platinum^®^ Tfi Exo(-) DNA Polymerase (Invitrogen), columns 5 and 6 with Pht Taq DNA Polimerase (Phoneutria) and columns 7 and 8 with Thermo Sequenase DNA Polimerase (GE).

FIGURE S3 Illustrated step-by-step guideline of the entire procedure to construct a chromosome-specific probe from a single microdissected chromosome: of the chromosome obtaining to its painting.

TABLE S1 Enzymes used for fluorescent labelling of DNA amplified from a single chromosome.
