# Supplementary figures and images for: Plant Chromosome-Specific Probes by Microdissection of a Single Chromosome: Is That a Reality?
Source: Front Plant Sci. 2020 Mar 25;11:334. doi: 10.3389/fpls.2020.00334 (PMC7113637; doi:10.3389/fpls.2020.00334)

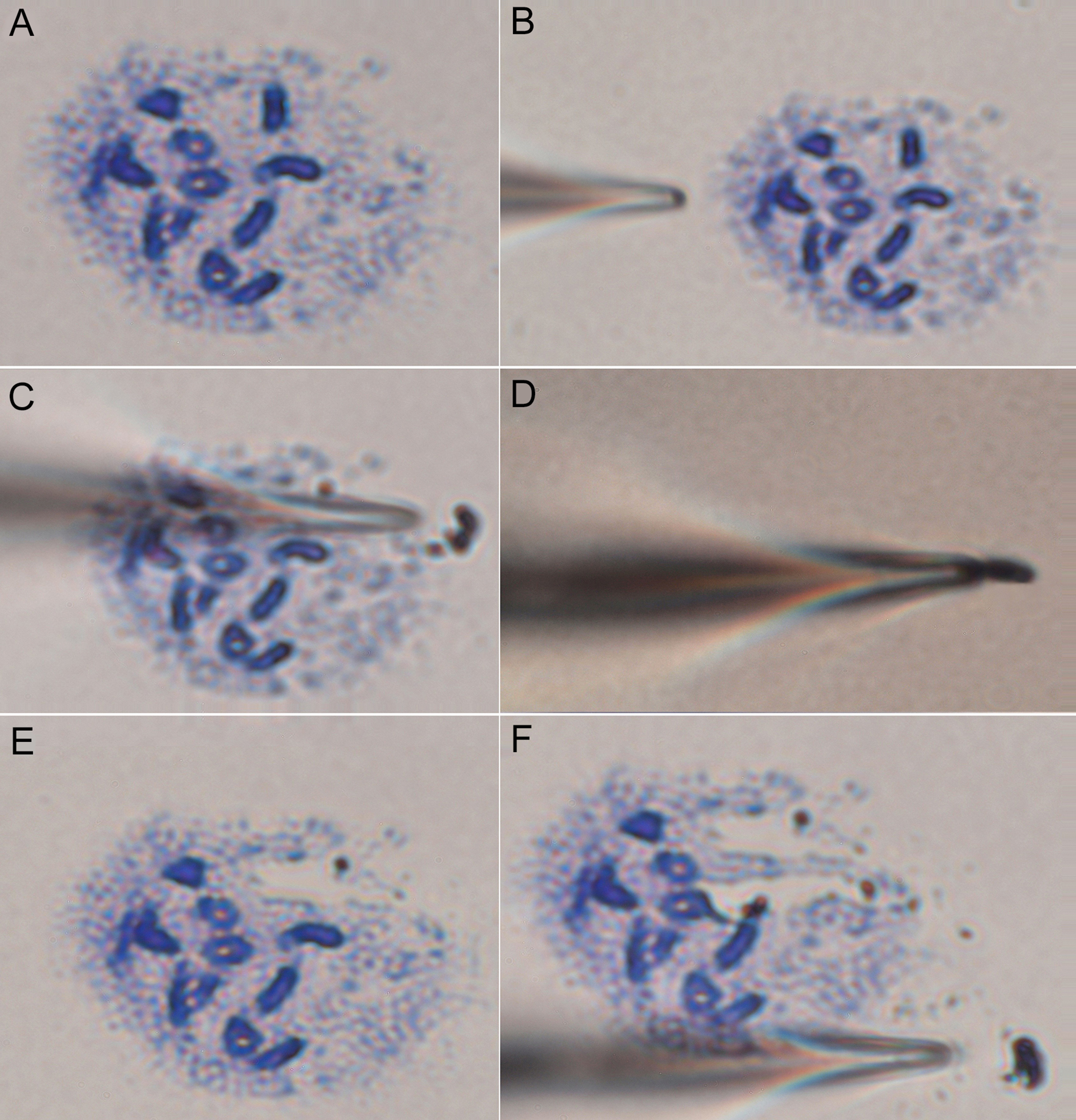

Supplement: FIGURE S1 — Microdissection of C. annuum bivalents at diplotene stage. (A) Bivalent at diplotene. (B) Glass microneedle for chromosome microdissection. Two focus planes: the slide and microneedle planes. (C) Microneedle removing a bivalent from the slide. (D) Chromosome adhered to the extremity of the microneedle. (E) Diplotene bivalent microdissected. (F) A second bivalent being removed showing reproducibility of the process. Each bivalent was placed in a different microtube, providing the second bivalent sample. The preparation was stained with a 5% Giemsa solution in a phosphate buffer (pH 6.8) for photo documentation only. [file Image_1.TIF]

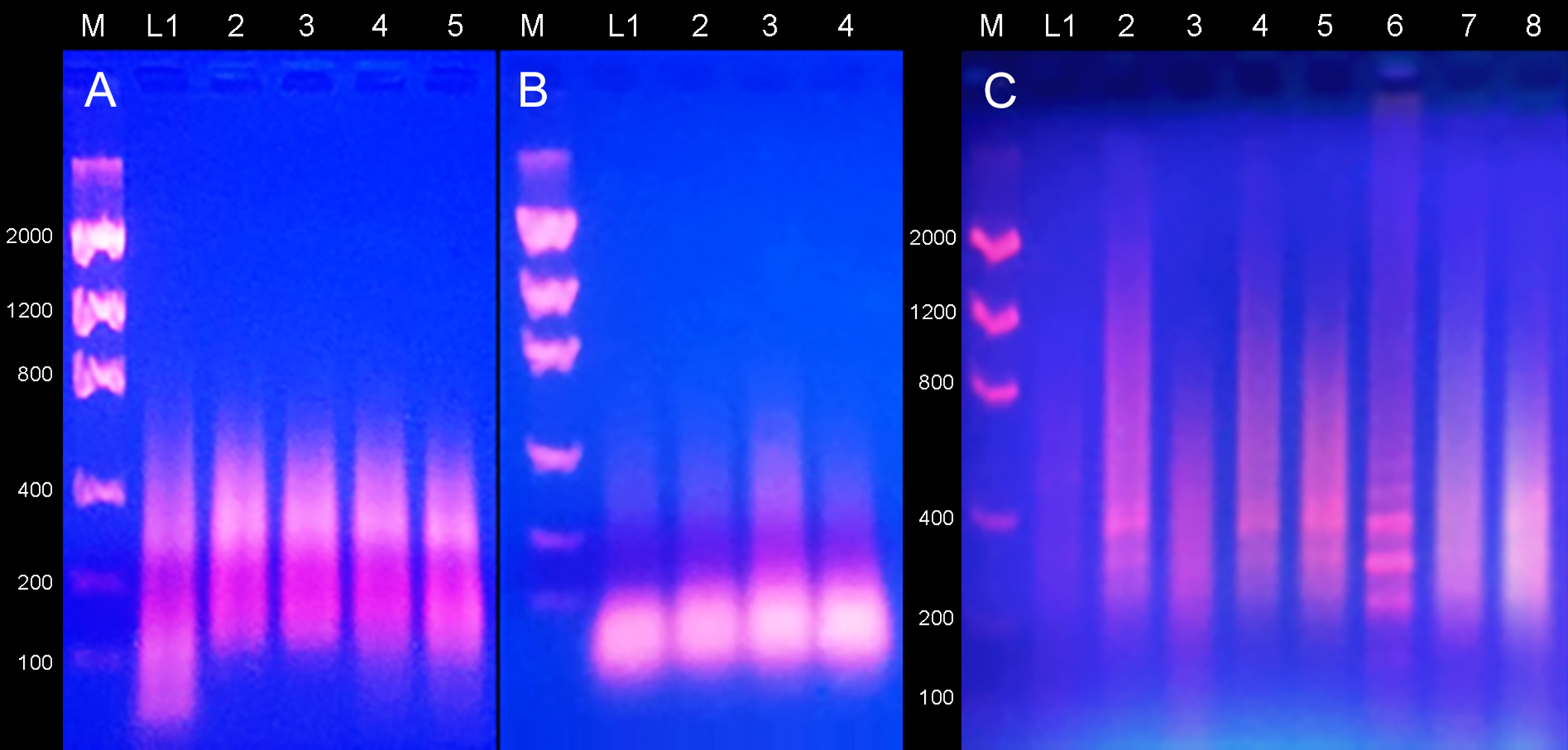

Supplement: FIGURE S2 — Representative DOP-PCR products analyzed in 1.5% agarose gel. Lines M represent the base pair marker and lines 1–8 the amplification products resulted of different microdissected chromosomes. (A) Human chromosomal DNA (columns 1–3) and Zea mays chromosomal DNA (columns 4 and 5) amplified with Thermo Sequenase DNA Polimerase (GE) and Platinum® Taq DNA Polymerase High Fidelity (Invitrogen), respectively. The fragment size ranged from 100 to 900 pb. (B) Human (columns 1 and 2) and Z. mays (columns 3 and 4) chromosomal DNA amplified with USB® Sequenase Version 2.0 DNA Polymerase, showing fragments size of 100–600 pb. (C) Human DNA fragments amplified (columns 2, 4, 6, and 8) and Z. mays (columns 1, 3, 5 and 7) labeled with Chroma Tide Alexa Fluor 5-dUTP. Columns 1 and 2 were labeled with AccuTaqTM LA DNA Polymerase (Sigma), columns 3 and 4 with Platinum® Tfi Exo(-) DNA Polymerase (Invitrogen), columns 5 and 6 with Pht Taq DNA Polimerase (Phoneutria) and columns 7 and 8 with Thermo Sequenase DNA Polimerase (GE). [file Image_2.TIF]
