# Supplementary material for: Plant Chromosome-Specific Probes by Microdissection of a Single Chromosome: Is That a Reality?
Source: Front Plant Sci. 2020 Mar 25;11:334. doi: 10.3389/fpls.2020.00334 (PMC7113637; doi:10.3389/fpls.2020.00334)
Supplement: FIGURE S3 — Illustrated step-by-step guideline of the entire procedure to construct a chromosome-specific probe from a single microdissected chromosome: of the chromosome obtaining to its painting. [file Presentation_1.PPTX]

## Slide 1
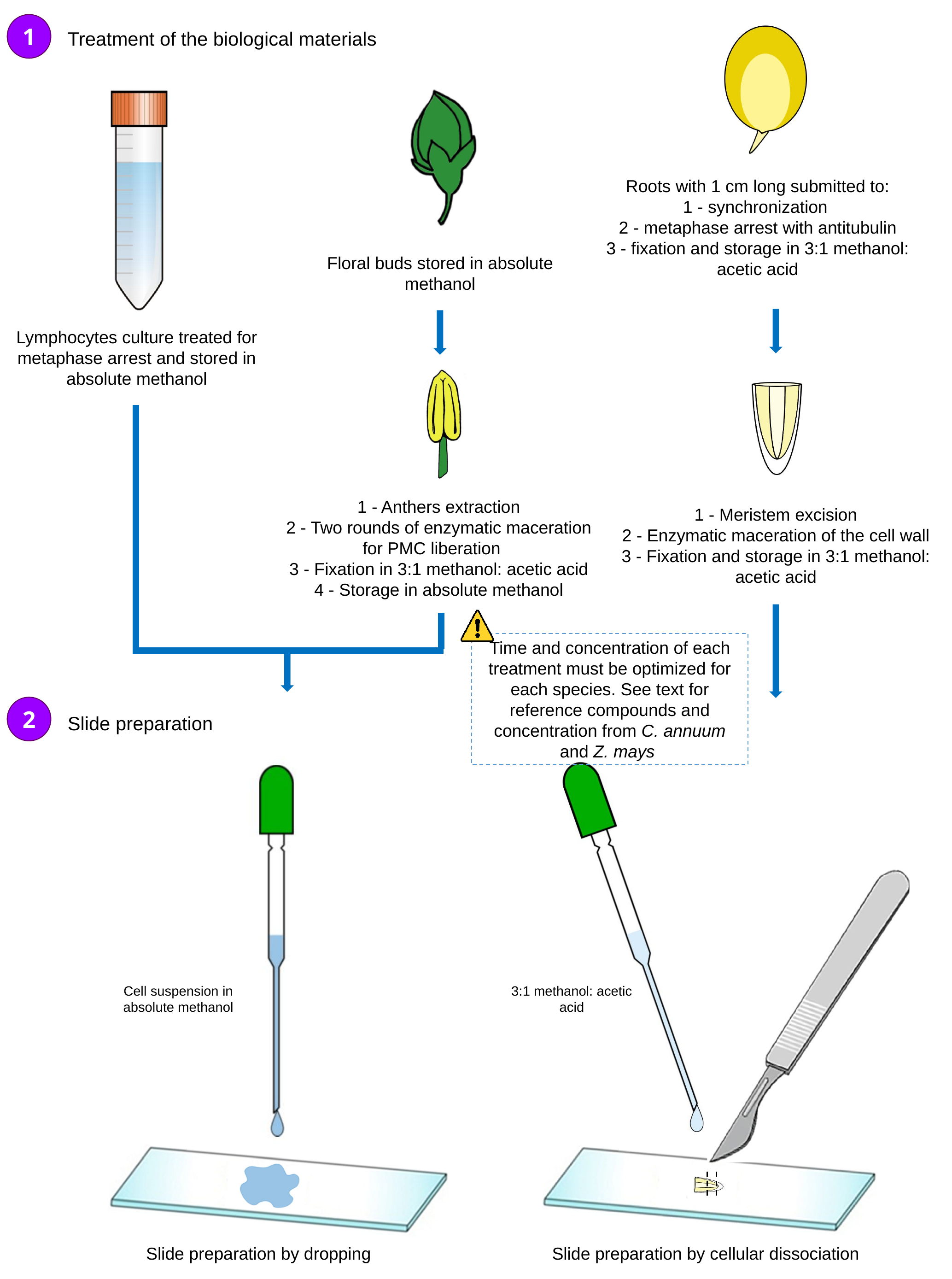

1
Treatment of the biological materials
Roots with 1 cm long submitted to:
1 - synchronization
2 - metaphase arrest with antitubulin
3 - fixation and storage in 3:1 methanol: acetic acid
Floral buds stored in absolute methanol
Lymphocytes culture treated for metaphase arrest and stored in absolute methanol
1 - Anthers extraction
2 - Two rounds of enzymatic maceration for PMC liberation
3 - Fixation in 3:1 methanol: acetic acid
4 - Storage in absolute methanol
1 - Meristem excision
2 - Enzymatic maceration of the cell wall
3 - Fixation and storage in 3:1 methanol: acetic acid
Time and concentration of each treatment must be optimized for each species. See text for reference compounds and concentration from C. annuum and Z. mays
2
Slide preparation
Cell suspension in absolute methanol
3:1 methanol: acetic acid
Slide preparation by dropping
Slide preparation by cellular dissociation

## Slide 2
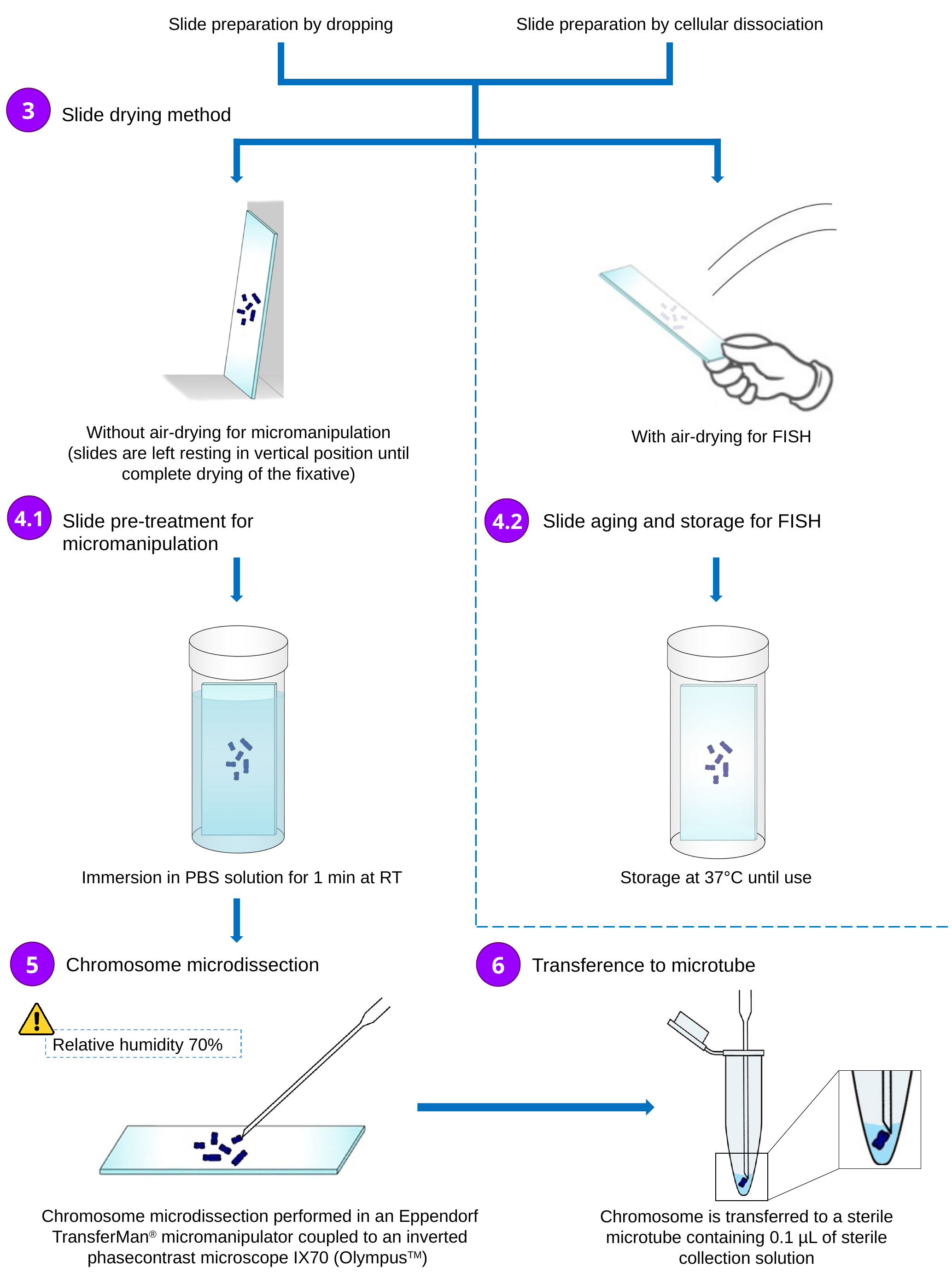

Slide preparation by dropping
Slide preparation by cellular dissociation
3
Slide drying method
Without air-drying for micromanipulation
(slides are left resting in vertical position until complete drying of the fixative)
With air-drying for FISH
4.1
4.2
Slide pre-treatment for micromanipulation
Slide aging and storage for FISH
Immersion in PBS solution for 1 min at RT
Storage at 37°C until use
5
6
Chromosome microdissection
Transference to microtube
Relative humidity 70%
Chromosome microdissection performed in an Eppendorf TransferMan® micromanipulator coupled to an inverted phasecontrast microscope IX70 (OlympusTM)
Chromosome is transferred to a sterile microtube containing 0.1 µL of sterile collection solution

## Slide 3
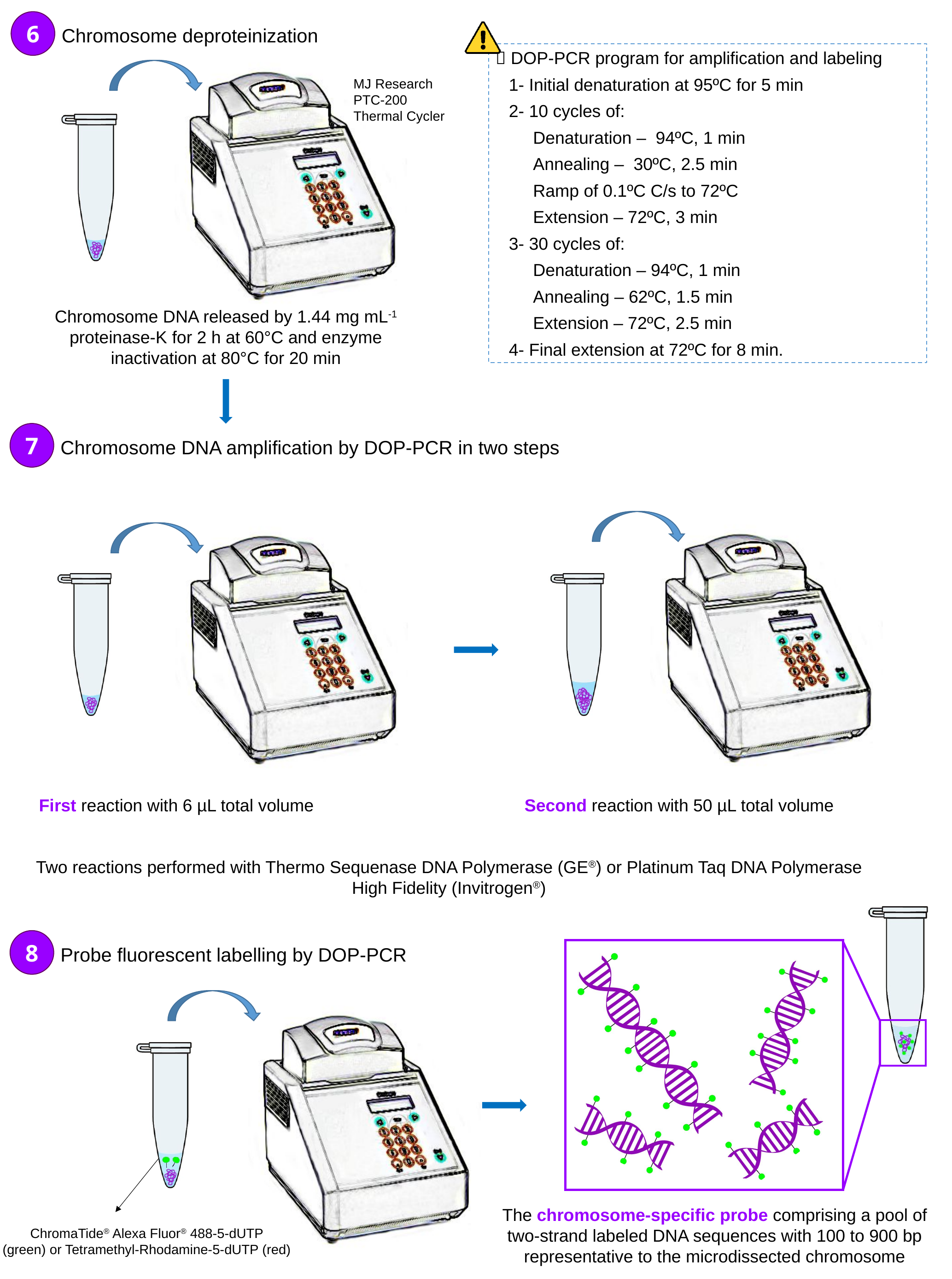

6
Chromosome deproteinization
 DOP-PCR program for amplification and labeling
1- Initial denaturation at 95ºC for 5 min
2- 10 cycles of:
 Denaturation – 94ºC, 1 min
 Annealing – 30ºC, 2.5 min
 Ramp of 0.1ºC C/s to 72ºC
 Extension – 72ºC, 3 min
3- 30 cycles of:
 Denaturation – 94ºC, 1 min
 Annealing – 62ºC, 1.5 min
 Extension – 72ºC, 2.5 min
4- Final extension at 72ºC for 8 min.
MJ Research PTC-200 Thermal Cycler
Chromosome DNA released by 1.44 mg mL-1 proteinase-K for 2 h at 60°C and enzyme inactivation at 80°C for 20 min
7
Chromosome DNA amplification by DOP-PCR in two steps
First reaction with 6 µL total volume
Second reaction with 50 µL total volume
Two reactions performed with Thermo Sequenase DNA Polymerase (GE®) or Platinum Taq DNA Polymerase High Fidelity (Invitrogen®)
8
Probe fluorescent labelling by DOP-PCR
The chromosome-specific probe comprising a pool of two-strand labeled DNA sequences with 100 to 900 bp representative to the microdissected chromosome
ChromaTide® Alexa Fluor® 488-5-dUTP
(green) or Tetramethyl-Rhodamine-5-dUTP (red)

## Slide 4
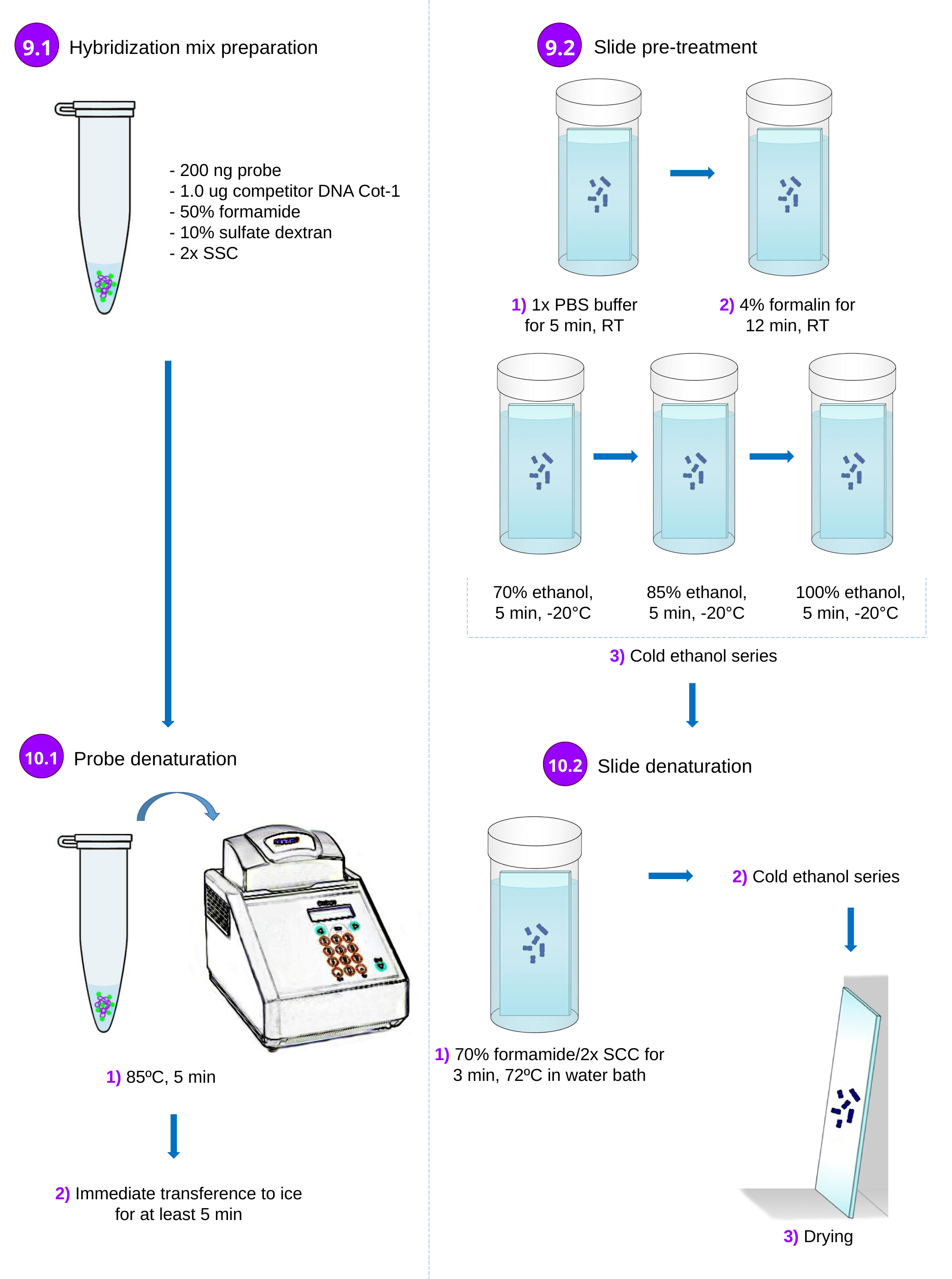

9.2
9.1
Slide pre-treatment
Hybridization mix preparation
- 200 ng probe
- 1.0 ug competitor DNA Cot-1
- 50% formamide
- 10% sulfate dextran
- 2x SSC
1) 1x PBS buffer for 5 min, RT
2) 4% formalin for
12 min, RT
70% ethanol,
5 min, -20°C
85% ethanol,
5 min, -20°C
100% ethanol,
5 min, -20°C
3) Cold ethanol series
Probe denaturation
10.1
Slide denaturation
10.2
2) Cold ethanol series
1) 70% formamide/2x SCC for
3 min, 72ºC in water bath
1) 85ºC, 5 min
2) Immediate transference to ice for at least 5 min
3) Drying

## Slide 5
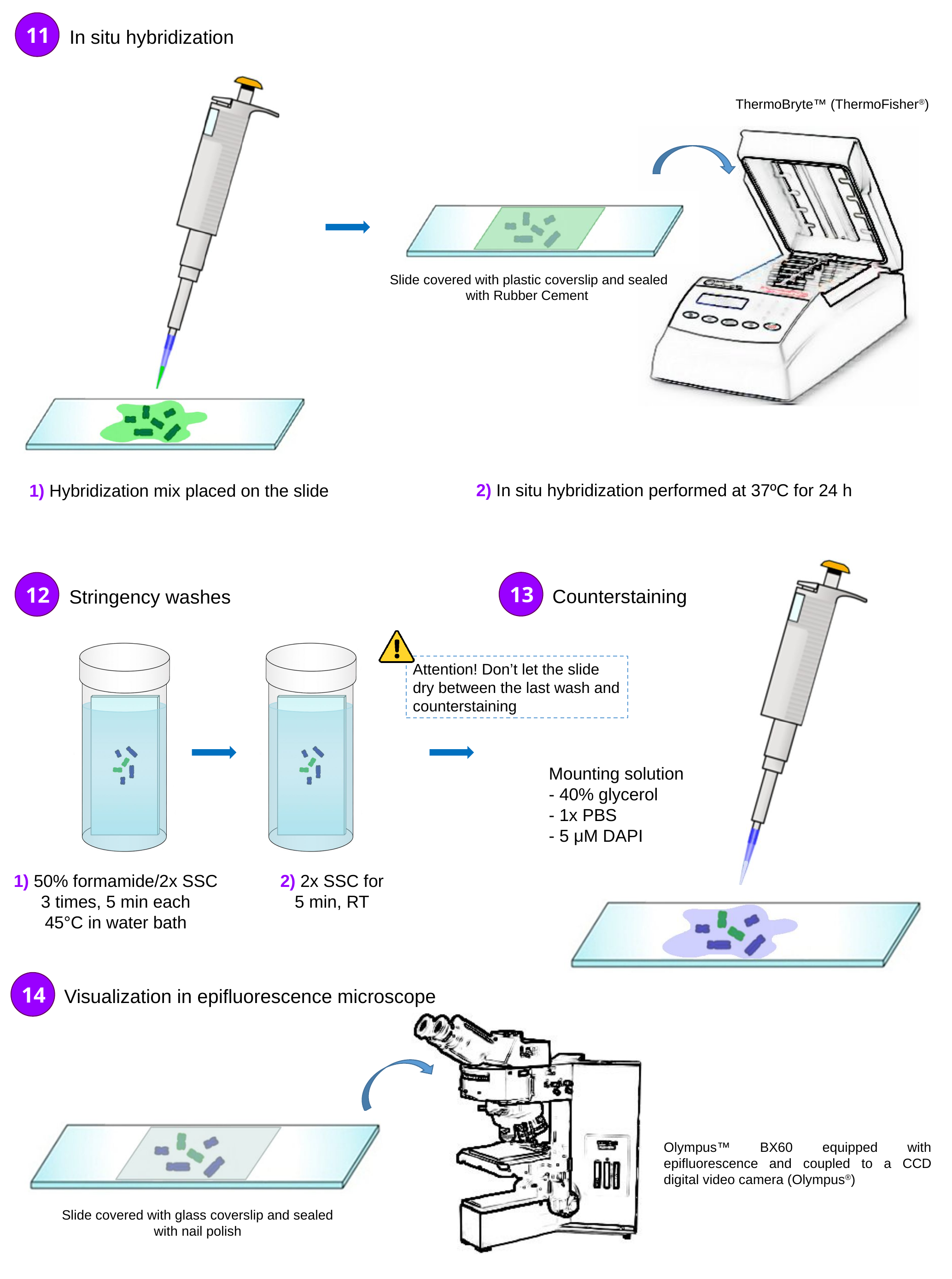

11
In situ hybridization
ThermoBryte™ (ThermoFisher®)
Slide covered with plastic coverslip and sealed with Rubber Cement
2) In situ hybridization performed at 37ºC for 24 h
1) Hybridization mix placed on the slide
13
12
Counterstaining
Stringency washes
Attention! Don’t let the slide dry between the last wash and counterstaining
Mounting solution
- 40% glycerol
- 1x PBS
- 5 μM DAPI
1) 50% formamide/2x SSC
3 times, 5 min each
45°C in water bath
2) 2x SSC for
5 min, RT
14
Visualization in epifluorescence microscope
Olympus™ BX60 equipped with epifluorescence and coupled to a CCD digital video camera (Olympus®)
Slide covered with glass coverslip and sealed with nail polish
