# Supplementary material for: Plant Chromosome-Specific Probes by Microdissection of a Single Chromosome: Is That a Reality?
Source: Front Plant Sci. 2020 Mar 25;11:334. doi: 10.3389/fpls.2020.00334 (PMC7113637; doi:10.3389/fpls.2020.00334)
Supplement: TABLE S1 — Enzymes used for fluorescent labelling of DNA amplified from a single chromosome. [file Table_1.docx]

**Table S1.** Enzymes used for fluorescent labelling of DNA amplified from a single chromosome.

| Enzyme | Particularity | Fluorochrome incorporation |
| --- | --- | --- |
| Thermo Sequenase DNA Polymerase (GE^®^) | High fidelity | yes* |
| Platinum^®^ Tfi Exo(-) DNA Polymerase (Invitrogen) | No 3’- 5’ or 5’ - 3’ correction | yes |
| AccuTaq^™^ LA DNA Polymerase (Sigma) | High fidelity | yes |
| Unitaq DNA Polymerase (Uniscience) | High fidelity | yes |
| Pht Taq DNA Polymerase (Phoneutria) | Low fidelity | yes |
| Hemo KlenTaq^®^ (BioLabs) | No 5’ - 3’ correction | yes |
| AmpliTaq^®^ DNA Polymerase (ThermoFisher) | High fidelity | no |
| Bst DNA Polymerase (BioLabs) | Displacement DNA amplification | no |
| Platinum^®^ Taq DNA Polymerase High Fidelity (Invitrogen) | High fidelity | no |
| Klenow Fragment (Takara) | No 5’ - 3’ correction | no |

*Higher relative fluorescence in agarose gel and FISH
